# Long-term monitoring of diversity and structure of two stands of an Atlantic Tropical Forest

**DOI:** 10.3897/BDJ.5.e13564

**Published:** 2017-07-19

**Authors:** Écio Souza Diniz, Warley Augusto Caldas Carvalho, Rubens Manoel Santos, Markus Gastauer, Paulo Oswaldo Garcia, Marco Aurélio Leite Fontes, Polyanne Aparecida Coelho, Aline Martins Moreira, Gisele Cristina Oliveira Menino, Ary Teixeira Oliveira-Filho

**Affiliations:** 1 Laboratory of Plant Ecology and Evolution, Department of Plant Biology, Federal University of Viçosa, Viçosa, Brazil; 2 Science Forest Department, Federal University of Lavras, Lavras, Brazil, Lavras, Brazil; 3 Instituto Tecnológico Vale, Belém, Brazil; 4 Instituto Federal de Educação, Ciência e Tecnologia Sul de Minas Gerais - Campus Muzambinho, Muzambinho, Brazil; 5 Science Forest Department, Federal University of Lavras, Lavras, Brazil; 6 Department of Biological Sciences, Lavras, Brazil; 7 Instituto Federal de Educação Ciência e Tecnologia Goiano - Campus de Rio Verde, Rio Verde, Brazil; 8 Federal University of Minas Gerais, Minas Gerais, Brazil

**Keywords:** Long-term monitoring, Succession, Diversity

## Abstract

**Background:**

This study aimed to report the long-term monitoring of diversity and structure of the tree community in a protected semideciduous Atlantic Forest in the South of Minas Gerais State, Southeast Brazil. The study was conducted in two stands (B and C), each with 26 and 38 10 m x 30 m plots. Censuses of stand B were conducted in 2000, 2005 and 2011, and stand C in 2001, 2006 and 2011. In both stands, the most abundant and important species for biomass accumulation over the inventories were trees larger than 20 cm of diameter, which characterize advanced successional stage within the forest.

**New information:**

The two surveyed stands within the studied forest presented differences in structure, diversity and species richness over the time.

## Introduction

The formation of the structure of tropical forests is governed by a wide range of factors (e.g. abiotic, biotic, neutral and natural or anthropogenic disturbance) (Guariguata and Kattan 2002, Hubbell 2001, Phillips et al. 2004). These factors lead to the establishment of a mosaic of forest patches of different ages (Guariguata and Kattan 2002, Oldemann 1990). These mosaics exhibit high environmental heterogeneity, which, associated with a combination of different floras, can lead to the formation of distinct local ecological patterns within a forest (Fortin and Dale 2005). However, the formation of these within-site differences is poorly understood, requiring further knowledge about community functioning. Tropical forests are both highly ecologically valuable and at great threat from disturbance. It is therefore vital to improve our knowledge of the mechanisms driving the formation of forest structure in order to protect and conserve the remaining areas of tropical forest.

Long-term research (community dynamics) is a vital tool in elucidating the causes of a wide range of ecological patterns and processes in plant communities (Phillips 1996). A better comprehension of events such as mortality, recruitment and ecological succession (Oliveira Filho et al. 2007, Saiter et al. 2011) will provide information about species’ behavior and community turnover, which may prove crucial to the success of projects aiming to conserve and restore vulnerable biomes from Tropical Forests.

Due to its ecological complexity, its endemic biodiversity and its threats by anthropogenic activities (e.g. agriculture, mining), the Brazilian Atlantic Forest is considered one of 25 hotspots of biodiversity (Mittermeier et al. 2005). Compared with its original cover, just 11.73 % (approximately 16,377,472 hectares) remains today, and few of these areas are adequately protected (Ribeiro et al. 2009). However, these few remaining protected sites provide a rare opportunity to monitor plant community dynamics, and to compare intact forest with heavily disturbed and fragmented stands. Therefore, this study aimed to report the long-term monitoring of diversity and tree community structure in two stands of a protected Atlantic Forest in the South of Minas Gerais State, Southeast Brazil. In addition, this paper also aimed to make data regarding forest dynamics from the studied site publicly available in order to encourage further research about the composition, diversity and structure of Atlantic Forests over time, thus contributing to the preservation of this threatened Biome.

## Project description

### Title

Population and community dynamics of a semideciduous forest from south Minas Gerais, Brazil

### Study area description

**Figure 1.**
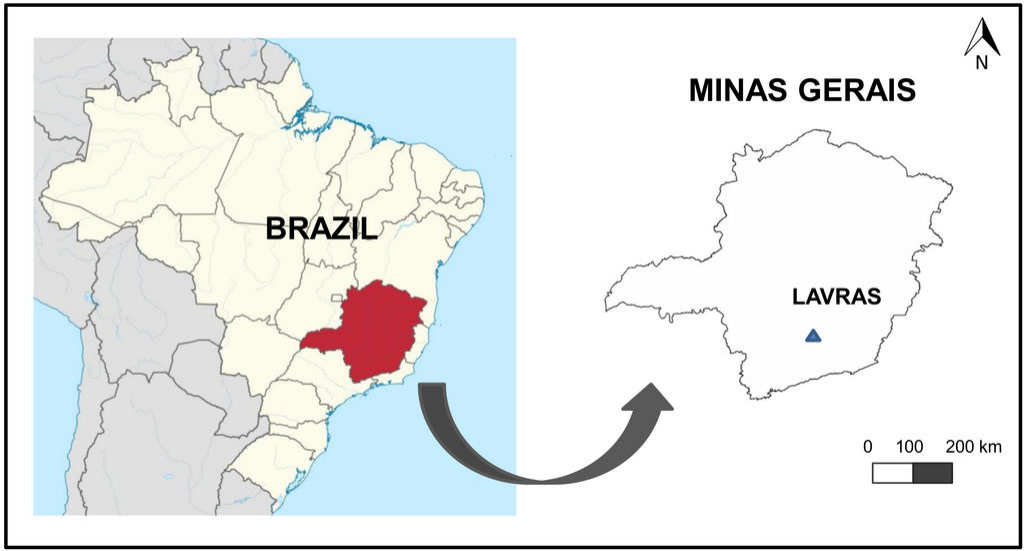
Location of the study site in Brazil and in Minas Gerais State.

The Parque Ecológico Quedas do Rio Bonito (PEQRB; “Falls of Nice River Ecological Park”) belongs to the Abraham Kasinski Foundation (FAK) and is located in the municipality of Lavras, in the Southern region of Minas Gerais State (21°19'S and 44°59'W), Brazil (Fig. 1), with elevations ranging from 950 and 1200 meters. (Oliveira Filho and Fluminhan-Filho 1999).

Within the park, the forest is distributed among valleys, on slopes and adjacent to watercourses, forming a heterogeneous environmental gradient with the occurence of typical mountain forest species such as *Protium widgrenii* Engl. (Burseraceae), *Clethra scabra* Pers. (Clethraceae), *Podocarpus sellowii* Klotzsch ex Endl. (Podocarpaceae), *Euplassa incana* (Klotzsch) I.M.Johnst. (Proteaceae) and *Drimys brasiliensis* Miers (Winteraceae) (Oliveira Filho and Fluminhan-Filho 1999). In addition, there are natural gaps and dense bamboo cover distributed throughout the forest analyzed in this study. The area of forest examined in this study is classified as Seasonal Semi-deciduous Upper-montane Forest (Oliveira Filho and Fluminhan-Filho 1999).

In 1999, 90% of the current area of the PEQRB was designated as protected in a decree from Lavras’ city hall, which includes the assignment to FAK. Before it was designated as a protected area, the PEQRB was subject to various disturbances. Disturbances such as free movement of cattle, logging for charcoal production and construction of housing and recreational areas (Dalanesi et al. 2004, Oliveira Filho and Fluminhan-Filho 1999).

## Sampling methods

### Sampling description

Investigation of floristic and structural composition of the tree community in PEQRB, its distribution over soil habitats and evaluation of the interaction of plants with environmental factors, were studied by Dalanesi et al. (2004). In the present study, the authors resurveyed two stands (called B and C), located 480 m apart, using plots of 300 m^2.^ Stand B was surveyed with 26 plots in 2000 comprising 0.78 hectares, which were kept and resurveyed in 2005 and 2011constituing its long-term monitoring. Stand C was surveyed in 2001 with 38 plots (1.14 hectares), then again in 2006 and 2011. The total cover of sampling area was 1.92 hectares.

**Figure 2.**
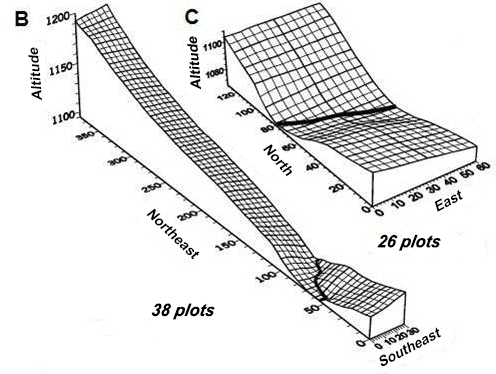
Surface grid of the sampled transects of each stand (B and C) studied in the forest of the PEQRB, in the municipality of Lavras, Minas Gerais State, Souheast of Brazil. The size of each plot in both stands was 30 x 10 meters. The spacing between grid lines is 5 meters. Adapted from Dalanesi et al. 2004.

The stands were arranged as transects (Fig. 2) perpendicular to the watercourse which bisects the park, and extends into two adjacent slopes, following principles outlined by Causton (1988). The samplings plots were contiguously allocated on each stand (Fig. 2). This sampling design of the was previously planned aiming to catch more environmental heterogeneity in the transects of both stands in order to analyze the relations among trees distribution with soil and topograghy (Dalanesi et al. 2004). All trees with diameter at breast height (DBH) greater than or equal to 4.99 cm were measured and permanently tagged. In the subsequent samplings, the surviving trees in both stands were remeasured, as well as all individuals which reached the criterion of inclusion. Additionally, the DBH of multi-stemmed trees was calculated as the square root of the sum of all squared stem DBH’s. Only multi-stemmed trees with DBH ≥ 4.99 cm were included in the survey as recruits.

Individuals were identified either in the field or through collection of samples of whole branches, leaves, and where possible, fruits. These samples were then compared with the existing collection present in herbarium “Herbário ESAL” of the Federal University of Lavras. In addition, samples were verified using appropriate literature and where necessary, specialists were consulted. The classification system followed that of the APG IV (Angiosperm Phylogeny Group) (2016) and we verified spellings and synonymous to the species through TNRS page (Taxonomic Name Resolution Service) Boyle et al. (2013).

**Data analyses**

**Species diversity and richness**

Species richness was compared between stands B and C stands using a Wilcoxon Rank Sum Test. The species diversity and evenness per stand were calculated by Shannon-Weaver (H) and Pielou (J) respectively, using the package “vegan” (Oksanen et al. 2017). To compare the total number of individuals and basal area per plot within each stand, a Nested ANOVA was carried out followed by a post-hoc test using the functions lmer and difflsmeans from the package lmerTest (Kuznetsova et al. 2016). The pairwise comparisons of number of individuals and basal area between B and C were conducted with a Two-Sample T-test of independent samples. All analyses mentioned above were carried out in R software version 3.3.0 (R Development Core Team 2016). Shannon-diversity between B and C was compared in Past software (Hammer et al. 2001) with a Hutcheson T-test (Hutcheson 1970).

**Tree Dynamics**

The changes in the tree community over the time were determined for each stand by calculating the annual mean rates of mortality (*M*) and recruitment (*R*) of individuals (based on species abundance) and basal area loss (*L*) and gain (*G*) according to Sheil et al. (1995), Sheil and May 1996 and Sheil et al. (2000). The time series were 11 years to stand B (surveyed in 2000, 2005 and 2011) and 10 years to stand C (surveyed in 2001, 2006 and 2011) constituting two periods to the calculation of the rates (B: 2000 and 2005, 2005 and 2011; C: 2001 and 2006, 2006 and 2011). In addition, the net rates of change in number of individuals and basal area were also calculated, based on the relationship between the abundance and basal area recorded in the first and most recent inventories. The net rate of change between inventories was calculated considering both abundance of trees (Ch_N_) and their basal area (Ch_AB_) Korning and Balslev 1994. To describe the rate of change in tree community, the turnover rates regarding both abundance (T_N_) and basal area (T_AB_), were calculated Phillips et al. 2004, Phillips 1996 for each stand.The turnover rates to number of individuals and basal area are respectively calculates as the averages of mortality, recruitment, and the loss and gain of basal area rates. . The difference between the number of dead and recruited trees was calculated using Poisson Counting (Zar 2010). This analysis was performed by calculating Exact Poisson Tests using R version 3.3.0 (R Development Core Team 2016) with the package “exactci” (Fay 2010).

**Tree Dynamics per diameter classes**

Diameter classes with increasing amplitudes were created (5-10 cm, >10-20 cm, >20-40 cm and >40-80 cm). This approach compensates for the sizeable decrease in abundance of the largest diameter individuals, which is a pattern commonly observed in tree diameter measurements and is characterized by the negative exponential distribution (Appolinário et al. 2005). To describe the temporal variations in each class, all individuals that underwent the following events: death, total *ingrowth* (inter-class imports through recruitment and growth) and total *outgrowth* (inter-class exports through growth and death) (Lieberman et al. 1985), were counted. The same procedure was used to calculate the absolute number of dead, absolute ingrowth (the absolute number of previously unrecorded individuals) and absolute outgrowth (the absolute number of individuals no longer present) per diameter class. To verify whether the frequency of the total number of dead trees within each stand (B: 2005 and 2011; C: 2006 and 2011) were dependent on diameter classes based on the frequencies expected from the second and last inventory diameter distribution, we used a G-Test of Goodness-of-Fit carried out with the package “DescTools” (Signorell 2016). The comparison between the total ingrowth and total outgrowth per class was carried out by calculating Exact Poisson Tests with the package “exactci” (Fay 2010). Both analyzes were carried out in R version 3.3.0.

To verify whether the frequency of dead trees within each stand (B: 2005 and 2011; C: 2006 and 2011) were dependent on diameter classes based on the frequencies expected from the second and last inventory diameter distribution

**Dynamics of the most abundant species**

The 10 most abundant species in each stand were selected and their mortality and recruitment rates were calculated. These species were also classified into regeneration guilds following the descriptions of Swaine and Whitmore (1988) and the field knowledge on the species of the authors of this study. This classification was used to aid in the deduction of the successional stage of the two forest stands. The difference between the absolute number of dead and recruits within the species was evaluated by Exact Poisson Tests in software R version 3.3.0 with the package “exactci” (Fay 2010).

## Geographic coverage

### Description

The Parque Ecológico Quedas do Rio Bonito (PEQRB; “Falls of Nice River Ecological Park”) is a particular protected with 235 hectares, located in the municipality of Lavras, in the Southern region of Minas Gerais State, with altitudes varying between 950 and 1200 metes.

### Coordinates

21°19' and Latitude; 44°59' and Longitude.

## Taxonomic coverage

### Description

**Results**

**Structure, diversity and species richness**

In stand B, the survey which took place in the year 2000 identified 1364 trees from 118 species (83 genera, 50 families); in 2005, 1313 trees in from 115 species were identified (80 genera, 48 families) and in 2011, 1251 trees from 106 species (75 genera, 46 families) were identified (Table 1). In stand C, in 2001, 1941 trees from 157 species (107 genera, 55 families) were identified; in 2006 1970 trees from 160 species were identified (107 genera, 55 families) and in 2011 1810 trees from 157 species were identified (105 genera, 53 families) (Table 1). The species richness was significantly different between stands B and C in each of the censuses (Table 2). In stand B the species richness decreased between 2000 and 2011 and in the stand C it was higher in 2006 (Table 2). The diversity in the stand B in all censuses was lower compared to stand C, according to Hutcheson T. comparisons (p<0.05; Table 2). Pielou’s evenness remained the same in stand B in 2000 and 2005, but decreased in 2001 (Table 2). On the other hand, stand C presented different evenness among all censuses with the highest degree of evenness detected in the 2011 census (Table 2).

In the stand B there was no significant difference in total basal area (F = 0.53, p = 0.593) or in total number of individuals (F = 0.23, p = 0.791) across all censuses. The same was evident in total basal area (F = 1.28, p = 0.279) and total number of individuals (F = 1.61, p = 0.201) in stand C (Table 2). The total number of individuals did not differ between stands across all of the censuses. There were significant differences in total basal area between stands across all of the censuses (Table 2).

The monitoring period of 11 years in stand B presented a decrease in the number of individuals per hectare, and an increase in basal area per hectare, from 2000 to 2011 (Table 2). The increase of basal area per hectare was also observed in stand B from 2001 to 2011, but the number of individuals per hectare decreased from 2006 to 2011 (Table 2).

**Tree Dynamics**

There was a decrease in abundance and increases in basal area during both intervals in stand B (2000-2005 and 2005-2011; Table 3). This clear loss of individuals was confirmed by the negative net change in both intervals, which was higher in the second interval (-0.96 % year^-1^), associated with an increase in mortality rate 3.02 % year^-1^) and a reduction in recruitment rate (1.63 % year^-1^). This was also reflected in a higher turnover (2.45 % year^-1^) in number of individuals. In fact, the number of dead trees was significantly higher compared to recruits in both intervals (2000 to 2005: Z = 2.94; *p* = 0.003; 2005 to 2011: Z = 3.65; *p* = 0.0003). There was a net increase in basal area in both intervals (2000-2005 = 1.04 % year^-1^ and 2005-2011 = 1.17 % year^-1^), which means that there is also an increase in biomass storage for the tree community during the 11 years of monitoring. This clear storage of biomass increased the turnover in basal area (3.14 % year^-1^) in the second interval, which was driven by the increase of gain in basal area rate (3.84% year^-1^).

In stand C, the number of individuals increased in the first interval (2001 to 2006) and decreased in the second (2006 to 2011). Basal area increased in both intervals (Table 3). In the period between 2006 and 2011, the net change rate was negative (-1.67 % year^-1^) resulting in a progressive loss of individuals. Consequently, the turnover rate increased (2.71 % year^-1^) and the number of dead individuals was significantly higher than recruits (Z = 7.11; *p* = < 0.0001). The biomass accumulation observed between 2001 and 2006 was elevated due to its positive net change (1.02 % year^-1^). However, the turnover in basal area during the second period (2006 to 2011) demonstrated a higher increase in biomass (3.08 % year^-1^).

**Tree Dynamics per diameter classes**

Stand B showed higher total outgrowth (2005 to 2011: *p* = < 0.0001, Table 2) in the 5-10 cm diameter class in both intervals, which was the result of a higher absolute number of outgrowth in relation to absolute ingrowth. In the >10 to 20 cm class in 2011, the total outgrowth was significantly higher than total ingrowth (*p* = < 0.0001). This was due to an increase in the number of mortalities and a reduction in the raw ingrowth (Table 4). Conversely, between 2000 and 2011, the >20 to 40 cm class exhibited an increase in abundance, where 2005 showed a higher total ingrowth than total outgrowth (*p* = 0.0057). The observed frequency of dead trees per diameter class in both 2005 and 2011 differed significantly from that expected under the null hypothesis, according to the G Test (*p* = < 0.0001), demonstrating that the frequency of mortality differs per diameter class and is dependent on which class it is in.

A progressive decrease of abundance in stand C was observed between 2001 and 2011 in the 5-10 cm class (Table 5). This was due to total outgrowth being significantly higher than total ingrowth in 2011 (p = < 0.0001) which was in turn caused by the increased mortality rate compared to 2006 (Table 3). In the >10 to 20 cm class the total outgrowth was higher than total ingrowth in both intervals (2006: p = <0.0001; 2011: p = 0.0395) as a result of the increased rates of mortality and raw outgrowth from 2006 to 2011. Conversely, in 2006 in the >20 to 40 cm class, total ingrowth was greater than total outgrowth (p = 0.0258). The frequency of dead trees in both periods was independent from the class in which it occurs, according to the G Test (2006 e 2011: p = < 0.0001).

**Dynamics of the most abundant species**

Among the 10 most abundant species in stand B (Table 6), four shade tolerant trees showed a significant pattern: *Amaioua intermedia*. *Copaifera langsdorfii*. *Faramea latifolia* and *Myrsine umbellata*. There was no observed net change in the number of individuals of *C. langsdorfii* and *F. latifolia* between 2000 and 2005, though both species did increase their numbers of individuals between 2005 and 2011 (0.67 % year^-1^ and 1.16 % year^-1^. respectively), while the net change rate in basal area decreased (0.99 % year^-1^ and 0.31 % year^-1^. respectively). On the other hand, *A. intermedia* (Table 6) exhibited a higher recruitment than mortality (2005: Z = 2.08. *P* = 0.03; 2011: Z = 3.46. *P* = 0.0005), which resulted in a higher number of individuals in both intervals. *Myrsine umbellata* was only amongst the most abundant species in 2000, and showed higher mortality rate than recruitment rate in 2011 (Z = 2.89. P = 0.03).

Of particular interest were the light-demanding species in stand B, specifically *Croton echinocarpus*, *Psychotria vellosiana*, *Pera glabrata* and *Tapirira obtusa*. *Psychotria. vellosiana* showed higher rates of mortality than recruitment in both intervals (2005: Z = 2.0040. *P* = 0.04; 2011: Z = 3.06. *P* = 0.0021). Its turnover rate in number of individuals was higher between 2000 and 2005 equating to 16.20 % year^-1^. Conversely, *P. glabrata* exhibited a progressive accumulation of biomass (basal area) from to 2000 to 2011, presenting higher turnover in basal area in 2011 (2.31 % year^-1^). *Tapirira obtusa* showed an increased mortality as recruitment decreased (Z = 2.89. *P* = 0.03; Z = 2.94. *P* = 0.03) in both periods. Having presented neither significant mortality nor recruitment rates, the pioneer species *C. echinocarpus* did not change significantly in abundance after the survey in 2000. The light-demanding species in stand C, *Miconia sellowiana*, *Myrcia splendens* and *Vochysia magnifica*, presented significant differences in the number of mortalities or recruitments. The first two were notable for high rates of mortality in 2011 (Z = 6.58. *P* = <0.0001 e Z = 7.92. *P* = <0.0001 respectively), whereas *V. magnifica* did not present recruitment and showed significant mortality in both intervals (2006: Z = 3.46. *P* = 0.0005 and 2011: Z = 3.68. *P* = 0.0002).

*Eremanthus erythropappus* also did not present recruitment in the second interval (between 2006 and 2011), in conjunction with higher rates of mortality (Z = 5.19. *P* = <0.0001) and only remained among the most abundant species in 2001. The shade tolerant *E. acutata* e *R. jasminoides* increased in basal area and abundance in both intervals (Table 6), but did not differ in mortality or recruitment.

## Usage rights

### Use license

Creative Commons Public Domain Waiver (CC-Zero)

## Data resources

### Data package title

PEQRB Population and Community Dynamics (2001-2011)

### Resource link

http://ipt.pensoft.net/

### Alternative identifiers

http://www.gbif.org/dataset/a8f27b2e-67a9-43cb-ad18-df43e0152c33; http://www.gbif.org/dataset/89f30480-54fd-4de2-ba51-c2249274add0

### Number of data sets

2

### Data set 1.

#### Data set name

peqrb_b_populationandcommunitydynamics_2000-2011

#### Data format

Darwin Core Archive DwC-A

#### Number of columns

40

#### Download URL

http://ipt.pensoft.net/resource?r=peqrb_b_populationandcommunitydynamics_2000-2011; http://www.gbif.org/dataset/a8f27b2e-67a9-43cb-ad18-df43e0152c33

#### Description

Occurrences, basal area and diameter at breast height of 1731 trees and treelets identified during three census distributed within 26 plots within stand B in the Parque Ecológico Quedas de Rio Bonito, Lavras, Minas Gerais, Brazil. Dataset consists of occurrence.txt (DwC-Attributes id, modified, language, rights, rightsHolder, bibliographicCitation, references, datasetName, basisOfRecord, occurrenceRemarks, eventDate, decimalLatitude, decimalLongitude, acceptedNameUsageID, parentNameUsageID, nameAccordingToID, scientificName, acceptedNameUsage, parentNameUsage, nameAccordingTo, higherClassification, kingdom, class, order, family, genus, subgenus, specificEpithet, infraSpecificEpithet, taxonRank, scientificNameAuthorship, nomenclaturalCode, taxonomicStatus), meta.xml, measurementOrFact.txt (continaining the DwC-Attributes id, measurementType, measurementUnit, measurementMethod, measurementValue, measurementRemarks), eml.xml, description.txt (containing the DwC-Attributes id, description, type, language). Please see http://rs.tdwg.org/dwc/ for details.

**Data set 1.** 

Column label	Column description
id	Occurrence identifier
modified	The most recent date-time on which the resource was changed.
rights	Information about who can access the resource or an indication of its security status.
rightsHolder	A person or organization owning or managing rights over the resource.
bibliographicCitation	A bibliographic reference for the resource as a statement indicating how this record should be cited (attributed) when used.
reference	A related resource that is referenced, cited, or otherwise pointed to by the described resource.
datasetName	The name identifying the data set from which the record was derived.
basisOfRecord	The specific nature of the data record.
eventDate	The date-time or interval during which an Event occurred. For occurrences, this is the date-time when the event was recorded.
decimalLatitude	The geographic latitude (in decimal degrees, using the spatial reference system given in geodeticDatum) of the geographic center of a Location.
decimalLongitude	The geographic longitude (in decimal degrees, using the spatial reference system given in geodeticDatum) of the geographic center of a Location.
acceptedNameUsageID	An identifier for the name usage (documented meaning of the name according to a source) of the currently valid (zoological) or accepted (botanical) taxon.
parentNameUsageID	An identifier for the name usage (documented meaning of the name according to a source) of the direct, most proximate higher-rank parent taxon (in a classification) of the most specific element of the scientificName.
nameAccordingToID	An identifier for the source in which the specific taxon concept circumscription is defined or implied.
scientificName	The full scientific name, with authorship and date information if known. When forming part of an Identification, this should be the name in lowest level taxonomic rank that can be determined.
acceptedNameUsage	The full name, with authorship and date information if known, of the currently valid (zoological) or accepted (botanical) taxon.
parentNameUsage	The full name, with authorship and date information if known, of the direct, most proximate higher-rank parent taxon (in a classification) of the most specific element of the scientificName.
nameAccordingTo	The reference to the source in which the specific taxon concept circumscription is defined or implied - traditionally signified by the Latin "sensu" or "sec." (from secundum, meaning "according to"). For taxa that result from identifications, a reference to the keys, monographs, experts and other sources should be given.
higherClassification	A list (concatenated and separated) of taxa names terminating at the rank immediately superior to the taxon referenced in the taxon record.
kingdom	The full scientific name of the kingdom in which the taxon is classified.
class	The full scientific name of the class in which the taxon is classified.
order	The full scientific name of the order in which the taxon is classified.
family	The full scientific name of the family in which the taxon is classified.
genus	The full scientific name of the genus in which the taxon is classified.
subgenus	The full scientific name of the subgenus in which the taxon is classified.
specificEpithet	The name of the first or species epithet of the scientificName.
intraspecificEpithet	The name of the lowest or terminal infraspecific epithet of the scientificName, excluding any rank designation.
taxonRank	The taxonomic rank of the most specific name in the scientificName.
scientificNameAutorship	The authorship information for the scientificName formatted according to the conventions of the applicable nomenclaturalCode.
nomenclaturalCode	The nomenclatural code (or codes in the case of an ambiregnal name) under which the scientificName is constructed. Recommended best practice is to use a controlled vocabulary.
taxonomicStatus	The status of the use of the scientificName as a label for a taxon.
description	Habitat Type
type	The nature or genre of the resource.
measurementType	The nature of the measurement, fact, characteristic, or assertion.
measurementValue	The units associated with the measurementValue.
measurementUnit	The units associated with the measurementValue.
measurementMethod	A description of or reference to (publication, URI) the method or protocol used to determine the measurement, fact, characteristic, or assertion.
measurementRemarks	Comments or notes accompanying the MeasurementOrFact.
language	A language of the resource.
occurrenceRemarks	Comments or notes about the Occurrence.

### Data set 2.

#### Data set name

peqrb_c_populationandcommunitydynamics_2001-2011

#### Data format

Darwin Core Archive DwC-A

#### Number of columns

40

#### Download URL

http://ipt.pensoft.net/resource?r=peqrb_c_populationandcommunitydynamics_2001-2011; http://www.gbif.org/dataset/89f30480-54fd-4de2-ba51-c2249274add0

#### Description

Occurrences, basal area and diameter at breast height of 1970 trees and treelets identified during three census distributed within 38 plots within stand C in the Parque Ecológico Quedas de Rio Bonito, Lavras, Minas Gerais, Brazil. Dataset consists of occurrence.txt (DwC-Attributes id, modified, language, rights, rightsHolder, bibliographicCitation, references, datasetName, basisOfRecord, occurrenceRemarks, eventDate, decimalLatitude, decimalLongitude, acceptedNameUsageID, parentNameUsageID, nameAccordingToID, scientificName, acceptedNameUsage, parentNameUsage, nameAccordingTo, higherClassification, kingdom, class, order, family, genus, subgenus, specificEpithet, infraSpecificEpithet, taxonRank, scientificNameAuthorship, nomenclaturalCode, taxonomicStatus), meta.xml, measurementOrFact.txt (continaining the DwC-Attributes id, measurementType, measurementUnit, measurementMethod, measurementValue, measurementRemarks), eml.xml, description.txt (containing the DwC-Attributes id, description, type, language). Please see http://rs.tdwg.org/dwc/ for details.

**Data set 2.** 

Column label	Column description
id	Occurrence identifier
modified	The most recent date-time on which the resource was changed.
rights	A legal document giving official permission to do something with the resource.
rightsHolder	A person or organization owning or managing rights over the resource.
bibliographicCitation	A bibliographic reference for the resource as a statement indicating how this record should be cited (attributed) when used.
reference	A related resource that is referenced, cited, or otherwise pointed to by the described resource.
datasetName	The name identifying the data set from which the record was derived.
basisOfRecords	The specific nature of the data record.
occurrenceRemarks	Comments or notes about the Occurrence.
eventDate	The date-time or interval during which an Event occurred. For occurrences, this is the date-time when the event was recorded.
decimalLatitude	The geographic latitude (in decimal degrees, using the spatial reference system given in geodeticDatum) of the geographic center of a Location.
decimalLongitude	The geographic longitude (in decimal degrees, using the spatial reference system given in geodeticDatum) of the geographic center of a Location.
acceptedNameUsageID	An identifier for the name usage (documented meaning of the name according to a source) of the accepted taxon.
parentNameUsageID	An identifier for the name usage (documented meaning of the name according to a source) of the direct, most proximate higher-rank parent taxon (in a classification) of the most specific element of the scientificName.
nameAccordingToID	An identifier for the source in which the specific taxon concept circumscription is defined or implied.
scientificName	The full scientific name, with authorship and date information if known. When forming part of an Identification, this is the name in lowest level taxonomic rank that can be determined. This term does not contain identification qualifications.
acceptedNameUsage	The full name, with authorship and date information if known, of the accepted botanical taxon.
parentNameUsage	The full name, with authorship and date information if known, of the direct, most proximate higher-rank parent taxon of the most specific element of the scientificName.
nameAccordingTo	The reference to the source in which the specific taxon concept circumscription is defined or implied.
higherClassification	A list (concatenated and separated) of taxa names terminating at the rank immediately superior to the taxon referenced in the taxon record.
kingdom	The full scientific name of the kingdom in which the taxon is classified.
class	The full scientific name of the class in which the taxon is classified.
order	The full scientific name of the order in which the taxon is classified.
family	The full scientific name of the family in which the taxon is classified.
genus	The full scientific name of the genus in which the taxon is classified.
subgenus	The full scientific name of the subgenus in which the taxon is classified.
specificEpithet	The name of the first or species epithet of the scientificName.
intraspecificEpithet	The name of the lowest or terminal infraspecific epithet of the scientificName, excluding any rank designation.
taxonRank	The taxonomic rank of the most specific name in the scientificName.
scientificNameAuthorship	The authorship information for the scientificName formatted according to the conventions of the applicable nomenclaturalCode.
nomenclaturalCode	The nomenclatural code (or codes in the case of an ambiregnal name) under which the scientificName is constructed.
taxonomicStatus	The status of the use of the scientificName as a label for a taxon.
description	Habitat type
type	The nature or genre of the resource. For Darwin Core, recommended best practice is to use the name of the class that defines the root of the record.
measurementType	The value of the measurement, fact, characteristic, or assertion.
measurementValue	The value of the measurement, fact, characteristic, or assertion.
measurementValue	The units associated with the measurementValue.
measurementUnit	A description of or reference to (publication, URI) the method or protocol used to determine the measurement, fact, characteristic, or assertion.
measurementMethod	A description of or reference to (publication, URI) the method or protocol used to determine the measurement, fact, characteristic, or assertion.
measurementRemarks	Comments or notes accompanying the MeasurementOrFac

## Additional information

**Discussion**

**Structure, diversity and species richness**

The diversity observed in stand C is amongst the highest observed in the region of this study (Pereira et al. 2006) and increased in the last census (Table 2). The location of the PEQRB in a transitional area between forests and cerrados (Brazilian savannah) certainly contributes to the enrichment of the local flora (Dalanesi et al. 2004). In addition to this transition between distinct vegetation types, the environmental heterogeneity (e.g. topography, soils properties and presence of forest edges) previously observed in PEQRB (Dalanesi et al. 2004) is reported as one of the main drivers of the species diversity in the remaining forests of the Alto Rio Grande region (Pereira et al. 2006).

On the other hand, the demonstrated reduction in values of species diversity and richness from 2000 to 2011 in stand B, concurrent with its decrease in evenness from 2005 to 2011 (Table 2), indicates the increase of dominance by a few species, which were found to increase in basal area and number of individuals (Brower and Zar 1990, Gurevitch et al. 2009). Thus, the increase in dominance reduces diversity and richness (Thorpe et al. 2011) demonstrating the natural successional advance which commonly promotes the exclusion of heliophiles and short life-span species (Swaine and Whitmore 1988).

**Tree dynamics**

The prevalence of basal area accumulation, in conjunction with reduction of abundance in stands B (in both intervals) and C (just in the first interval), gives rise to a mature forest. This is indicative of an advanced successional stage in both stands of the studied forest (Oldemann 1990, Oldeman 1989). In sites where clearcutting has taken place, successional change inevitably leads to a phase of self-thinning, whereby overcrowding creates high levels of mortality (Gomes et al. 2003, Oldemann 1990, Oliveira-Filho et al. 1997). Although PEQRB has a history of clearcutting and burning, there is no precise data or records of the exact location relative to the monitored stands, prior to the most recent survey carried out for this study in 2011. However, this pattern of biomass gain and local abundance decrease in the community is common in tropical forests which are protected from intense man-made disturbance, thus allowing the natural advance of succession (Chazdon 2008, Guariguata and Ostertag 2001, Letcher and Chazdon 2009, Letcher 2010, Muscarella et al. 2016). When natural succession occurs, tree interactions increase and events, such as competitive exclusion from the larger and taller canopy trees suppressing the smaller ones present in the understory, achieve higher importance in community assembly (Chazdon 2008, Letcher et al. 2012, Letcher 2010).

The transition of dynamic patterns observed in stand C (1° survey: simultaneous increase of abundance and basal area; 2° survey: increase of basal area and decrease of abundance) is possibly caused by gap formation (Whitmore 1988, Silva et al. 2011) and the dynamic balance between mortality and recruitment (Phillips 1996). These are some of the main factors responsible for the change in tree community structure in conserved and protected sites such as PEQRB (Oliveira-Filho et al. 1997, Letcher 2010, Phillips et al. 2004, Stephenson and Mantgem 2005). Another possibility could be that events of mortality and recruitment may appear to act at different intensities at a local level. It is possible that these incidents may be just part of the natural dynamics of a stable community (Felfili 1995, Korning and Balslev 1994, Phillips and Gentry 1994, Saiter et al. 2011, Sheil et al. 2000). However, the events of mortality and recruitment are often associated with stochastic factors (Hubbell 2001, Laurance et al. 2011) and therefore difficult to interpret in short-term temporal scales (Oldeman 1989).

The location of the stands in two different locations within the forest in PQERB can be also considered relevant as an explanatory factor for the observed rates of mortality and recruitment, because these stands encompass various ecological units of vegetation with distinct ages of formation, leading to a heterogeneous forest with multiple successional stages (Oldemann 1990, Phillips 1996).

**Tree Dynamics per diameter classes**

The J-inverted distribution of individuals per diameter class is typical of many tropical forests and has been reported to be the case in other forests in the region of the studied site (Appolinário et al. 2005, Guimarães et al. 2008, Oliveira Filho et al. 2007). This study showed that the forests of the PEQRB also conform to this distribution. The accentuated reduction of abundance in the smaller diameter class (5-10 cm) is implicit in J-inverted distribution. This happens because in this class the individuals are very size and density dependent (Farrior et al. 2016), meaning individuals are less competitive (Weiner 1990) and are more sensitive to natural disturbances, such as the fall of a big tree (Lieberman et al. 1985, Swaine et al. 1987, Appolinário et al. 2005, Guariguata and Ostertag 2001, Letcher 2010). Conversely, the increase in abundance of individuals > 10 cm in stand B and > 20 cm in stand C corroborate the maturing status of the tree component of the forest in PQERB (Swaine et al. 1987, Oldeman 1989, Phillips et al. 2004).

**Dynamics of the most abundant species**

Shade-tolerant species are able to develop under a closed canopy and require little sunlight. Conversely, the light-demanding species need higher sunlight incidence in order to develop and establish in a site. Both types of species occur in mature tropical forests in ongoing successional advance (Swaine and Whitmore 1988) and were more abundant compared to the pioneers, which were not among the most abundant ones in all inventories. One such example is *Croton echinocarpus*, which presented mostly bigger trees (> 10 cm) in the first two inventories. In a similar way, *Myrsine umbellata* was only among the most abundant species in the survey in 2000, probably as a result of the decreasing abundance of the individuals from 5-10 cm and similar abundance of individuals > 10 cm, in both intervals. During successional advance and in the exclusion stem phase (Letcher 2010), few trees survive the intensification of competition, caused by the biomass accumulation and higher crown of more competitive species, for light (Farrior et al. 2016). Smaller, less competitive trees (Coomes and R.B 2007) such as halophytic species with short life cycles die (Oldeman 1989, Chazdon et al. 2010).

The importance of light-demanding and shade-tolerant species in the community is demonstrated by their progressive increase in abundance and basal area. This confirms their important role in the formation of community structure and successional advance (Felfili 1995, Oliveira Filho et al. 2004). Stand B differs from stand C in that there was a larger number of light-demanding species and fewer shade-tolerant species. This finding possibly reflects the presence of more gaps in stand C than B, allowing halophytic species to remain in the forest interior (Lieberman et al. 1985, Pereira et al. 2006) as the forest develops towards the most advanced successional stages (Oldeman 1989, Guariguata and Ostertag 2001, Condit et al. 2002).

**General conclusions on forest dynamics and conservation**

The higher diversity in stand C, plus the diversity in stand B, indicates high alpha diversity within the PEQRB forest. This highlights the importance of the protection of biotic resources, and also supports the demand for further research to understand underlying determinants of this diversity. Despite the differences found in the structural dynamics within the studied forest, the basal area increased in both stands indicating biomass accumulation. This is a key factor in ecosystem services, such as the amplification of carbon stock through biomass gain. These two points show the importance of the protection of Atlantic Forests and that studies like this are important so that we may better understand the drivers of forest dynamics. Thus, the continued monitoring of this study site is necessary to further refine the mechanisms underlying tree dynamics.

## Figures and Tables

**Table 1.** Families and species: presence and absence in the stands B and C of the Semideciduous Atlantic Forest at Parque Ecológico Quedas do Rio Bonito (PEQRB), municipality of Lavras, South of Minas Gerais State, Southeast Brazil.

**Families/Species**	**Presence/Absence**					
	**B**			**C**		
	**2000**	**2005**	**2011**	**2001**	**2006**	**2011**
** Anacardiaceae **						
*Tapirira guianensis* Aubl.	x	x	x	x	x	x
*Tapirira obtusa* (Benth.) J.D.Mitchell	x	x	x	x	x	x
** Annonaceae **						
*Annona cacans* Warm.	x	x	x	x	x	x
*Annona neolaurifolia* H.Rainer	-	-	-	x	x	x
*Annona neosericea* H.Rainer	-	-	-	x	x	x
*Annona sylvatica* A.St.-Hil.	-	-	-	x	x	x
*Guatteria australis* A.St.-Hil.	x	x	x	x	x	x
*Xylopia brasiliensis* Spreng.	x	x	x	x	x	x
** Apocynaceae **						
*Aspidosperma australe* Müll.Arg.	x	x	x	x	x	x
*Aspidosperma olivaceum* Müll.Arg.	x	x	x	x	x	x
*Aspidosperma spruceanum* Benth. ex Müll.Arg.	-	-	-	x	x	x
** Aquifoliaceae **						
*Ilex cerasifolia* Reissek	x	x	x	x	x	x
*Ilex conocarpa* Reissek	x	x	-	x	x	x
*Ilex paraguariensis* A.St.-Hil.	x	x	x	-	-	-
*Ilex sapotifolia* Reissek	x	x	x	-	-	-
** Araliaceae **						
*Dendropanax cuneatus* (DC.) Decne. & Planch.	-	-	-	x	x	x
*Schefflera calva* (Cham.) Frodin & Fiaschi	-	-	-	x	x	x
** Arecaceae **						
*Geonoma pohliana* Mart.	-	-	-	x	x	x
*Geonoma schottiana* Mart.	x	x	x	x	x	x
*Syagrus flexuosa* (Mart.) Becc.	-	-	-	x	x	x
** Asteraceae **						
*Austrocritonia velutina* (Gardner) R.M.King & H.Rob.	-	x	-	-	-	-
*Baccharis oblongifolia* (Ruiz & Pav.) Pers.	x	x	-	-	-	-
*Eremanthus erythropappus* (DC.) MacLeish	x	x	x	x	x	x
*Piptocarpha axillaris* (Less.) Baker	-	-	-	x	x	x
*Piptocarpha macropoda* Baker	-	-	-	x	x	x
*Vernonanthura divaricata* (Spreng.) H.Rob.	-	-	-	x	x	x
** Bignoniaceae **						
*Handroanthus serratifolius* (Vahl) S.O.Grose	-	-	-	x	x	x
*Jacaranda macrantha* Cham	x	x	x	x	x	x
*Jacaranda puberula* Cham.	-	-	-	x	x	x
** Boraginaceae **						
*Cordia sellowiana* Cham.	x	x	x	x	x	x
** Burseraceae **						
*Protium heptaphyllum* (Aubl.) Marchand	-	x	-	-	-	-
*Protium spruceanum* (Benth.) Engl.	x	x	x	-	x	-
*Protium widgrenii* Engl.	x	x	x	x	x	x
** Celastraceae **						
*Maytenus communis* Reissek	x	x	x	x	x	x
*Maytenus evonymoides* Reissek	-	-	-	x	x	x
*Maytenus gonoclada* Mart.	x	x	x	x	x	x
*Maytenus salicifolia* Reissek	x	x	x	x	x	x
*Salacia elliptica* (Mart. ex Schult.) G.Don	x	x	x	x	x	x
** Chrysobalanaceae **						
*Hirtella hebeclada* Moric. ex DC.	x	x	x	-	-	-
** Clethraceae **						
*Clethra scabra* Pers.	x	x	x	x	x	x
** Clusiaceae **						
*Calophyllum brasiliense* Cambess.	-	-	-	x	x	x
*Garcinia gardneriana* (Planch. & Triana) Zappi	-	-	-	x	x	x
*Kielmeyera lathrophyton* Saddi	x	-	-	-	-	-
** Combretaceae **						
*Terminalia glabrescens* Mart.	-	-	-	x	x	-
** Connaraceae **						
*Connarus regnellii* G.Schellenb.	-	-	-	x	x	x
** Cunoniaceae **						
*Lamanonia ternata* Vell.	x	x	-	x	-	-
** Cyatheaceae **						
*Alsophila sternbergii* (Pohl ex Sternb.) Conant	x	-	-	-	-	-
*Cyathea delgadii* Sternb.	x	x	x	-	-	-
*Cyathea gardneri* Hook.	x	x	x	-	-	-
*Cyathea phalerata* Mart.	x	x	x	x	x	x
** Elaeocarpaceae **						
*Sloanea hirsuta* (Schott) Planch. ex Benth	x	x	x	x	x	x
** Euphorbiaceae **						
*Alchornea glandulosa* Poepp. & Endl.	-	-	-	x	x	x
*Alchornea triplinervia* (Spreng.) Müll.Arg.	x	x	x	x	x	x
*Croton echinocarpus* Müll.Arg.	x	x	x	x	x	x
*Croton floribundus* Spreng.	x	x	x	x	x	x
*Pera glabrata* (Schott) Poepp. ex Baill.	x	x	x	x	x	x
*Sapium glandulosum* (L.) Morong	-	-	-	x	x	x
*Sebastiania commersoniana* (Baill.) L.B.Sm. & Downs	x	x	x	x	x	x
** Fabaceae Caesalpinioideae **						
*Cassia ferruginea* (Schrad.) Schrad. ex DC.	-	-	-	x	x	x
*Copaifera langsdorffii* Desf.	x	x	x	x	x	x
*Senna macranthera* (DC. ex Collad.) H.S.Irwin & Barneby	-	-	-	-	x	x
*Tachigali rugosa* (Mart. ex Benth.) Zarucchi & Pipoly	x	x	x	x	x	x
** Fabaceae Faboideae **						
*Dalbergia frutescens* (Vell.) Britton	x	x	-	x	x	-
*Dalbergia villosa* (Benth.) Benth.	x	x	x	x	x	x
*Erythrina falcata* Benth.	-	-	-	x	-	x
*Machaerium nictitans* (Vell.) Benth.	-	-	-	x	x	x
*Machaerium villosum* Vogel	x	x	x	x	x	x
*Platycyamus regnellii* Benth.	-	-	-	x	x	x
** Fabaceae Mimosoideae **						
*Inga ingoides* (Rich.) Willd.	-	-	-	x	x	x
*Inga marginata* Willd.	-	-	-	x	x	x
*Inga striata* Benth.	-	-	-	x	x	x
*Inga vera* Willd.	-	-	-	x	x	x
*Leucochloron incuriale* (Vell.) Barneby & J.W.Grimes	x	x	x	x	x	x
*Piptadenia gonoacantha* (Mart.) Macbr.	-	-	-	x	x	x
*Pseudopiptadenia leptostachya* (Benth.) Rausch.	-	-	-	x	x	x
** Humiriaceae **						
*Humiriastrum glaziovii* (Urb.) Cuatrec.	x	-	-	-	-	-
*Sacoglottis mattogrossensis* Malme	x	x	x	-	-	-
** Hypericaceae **						
*Vismia brasiliensis* Choisy	x	x	x	x	x	x
** Lacistemataceae **						
*Lacistema hasslerianum* Chodat	x	x	x	x	x	x
** Lamiaceae **						
*Aegiphila verticillata* Vell.	-	-	-	-	-	x
*Vitex megapotamica* (Spreng.) Moldenke	x	x	x	x	x	x
*Vitex polygama* Cham.	x	x	x	x	x	x
** Lauraceae **						
*Aniba firmula* (Nees & Mart.) Mez	x	x	x	-	-	-
*Cinnamomum glaziovii* (Mez) Kosterm.	x	x	x	x	-	-
*Cryptocarya aschersoniana* Mez	x	x	x	x	x	x
*Endlicheria paniculata* (Spreng.) J.F.Macbr.	-	-	-	x	-	-
*Nectandra grandiflora* Nees	x	x	x	x	x	x
*Nectandra megapotamica* (Spreng.) Mez	x	x	x	-	-	-
*Nectandra nitidula* Nees	-	-	-	-	x	x
*Nectandra oppositifolia* Nees	x	x	x	x	x	x
*Ocotea aciphylla* (Nees & Mart.) Mez	x	x	x	x	-	-
*Ocotea brachybotrya* (Meisn.) Mez	x	-	-	x	-	-
*Ocotea corymbosa* (Meisn.) Mez	x	x	x	x	x	x
*Ocotea diospyrifolia* (Meisner) Mez	-	-	-	x	x	x
*Ocotea indecora* (Schott) Mez	-	-	-	x	x	x
*Ocotea odorifera* (Vell.) Rohwer	x	x	x	x	x	x
*Ocotea pulchella* (Nees & Mart.) Mez	x	x	x	x	x	x
*Persea major* L.E.Kopp	x	x	x	x	x	x
*Persea willdenovii* Kosterm.	x	x	-	x	x	-
** Lecythidaceae **						
*Cariniana legalis* (Mart.) Kuntze	-	-	-	x	x	x
** Lythraceae **						
*Lafoensia glyptocarpa* Koehne	-	-	-	-	-	x
*Lafoensia pacari* A.St.-Hil.	x	x	x	x	x	x
** Magnoliaceae **						
*Magnolia ovata* (A.St.-Hil.) Spreng.	x	-	-	-	-	-
** Malpighiaceae **						
*Byrsonima laxiflora* Griseb.	x	x	x	x	x	x
*Heteropterys byrsonimifolia* A.Juss.	x	x	x	-	-	-
** Malvaceae **						
*Luehea grandiflora* Mart. & Zucc.	x	x	x	-	-	-
** Melastomataceae **						
*Leandra melastomoides* Raddi	x	-	-	-	-	-
*Miconia chartacea* Triana	x	x	x	x	x	x
*Miconia cinnamomifolia* (DC.) Naudin	x	x	x	x	x	x
*Miconia latecrenata* (DC.) Naudin	-	-	-	-	x	x
*Miconia sellowiana* Naudin	x	x	x	x	x	x
*Tibouchina stenocarpa* (DC.) Cogn.	-	-	-	x	x	x
** Meliaceae **						
*Cabralea canjerana* (Vell.) Mart.	x	x	x	x	x	x
*Cedrela fissilis* Vell.	-	-	-	x	x	x
*Guarea macrophylla* Vahl	-	-	-	-	x	-
*Trichilia elegans* A.Juss.	-	-	-	x	x	x
** Monimiaceae **						
*Mollinedia argyrogyna* Perkins	x	x	x	x	x	x
** Moraceae **						
*Sorocea bonplandii* (Baill.) W.C.Burger, Lanj. & Wess.Boer	x	x	x	x	x	x
** Myrtaceae **						
*Blepharocalyx salicifolius* (Kunth) O.Berg	x	x	x	x	x	x
*Calyptranthes clusiifolia* (Miq.) O.Berg	x	x	x	x	x	x
*Calyptranthes widgreniana* O.Berg	x	x	x	-	-	x
*Campomanesia guaviroba* (DC.) Kiaersk.	-	-	-	x	x	x
*Campomanesia guazumifolia* (Cambess.) O.Berg	-	-	-	x	x	x
*Eugenia acutata* Miq.	-	-	-	x	x	x
*Eugenia dodonaeifolia* Cambess.	-	-	-	x	x	x
*Eugenia florida* DC.	-	-	-	x	x	x
*Eugenia handroana* D.Legrand	x	x	x	-	-	-
*Eugenia hyemalis* Cambess.	x	x	x	x	x	x
*Marlierea racemosa* (Vell.) Kiaersk.	x	x	x	x	x	x
*Myrceugenia miersiana* (Gardner) D.Legrand & Kausel	-	-	-	x	x	x
*Myrcia eriocalyx* DC.	x	x	x	x	-	-
*Myrcia guianensis* (Aubl.) DC.	-	-	-	x	x	x
*Myrcia hebepetala* DC.	x	x	x	x	x	x
*Myrcia obovata* (O.Berg) Nied.	x	x	x	x	x	x
*Myrcia pulchra* (O.Berg) Kiaersk.	x	x	x	-	-	-
*Myrcia splendens* (Sw.) DC.	x	x	x	x	x	x
*Myrcia tomentosa* (Aubl.) DC.	-	-	-	x	x	x
*Myrcia venulosa* DC.	x	x	x	x	x	x
*Myrciaria floribunda* (H.West ex Willd.) O.Berg	x	x	x	x	x	x
*Myrceugenia rufescens* (DC.) D.Legrand & Kausel	-	-	x	-	-	-
*Pimenta pseudocaryophyllus* (Gomes) Landrum	x	x	-	x	x	x
*Siphoneugena densiflora* O.Berg	x	x	x	x	x	x
*Siphoneugena widgreniana* O.Berg	-	-	-	x	x	x
*Syzygium jambos* (L.) Alston	-	-	-	x	x	x
** Nyctaginaceae **						
*Guapira opposita* (Vell.) Reitz	x	x	x	x	x	x
** Olacaceae **						
*Heisteria silvianii* Schwacke	x	x	x	x	x	x
** Opiliaceae **						
*Agonandra excelsa* Griseb.	-	-	-	x	x	x
** Pentaphylacaceae **						
*Ternstroemia brasiliensis* Cambess.	x	x	x	x	x	x
** Piperaceae **						
*Piper cernuum* Vell.	-	-	-	x	x	x
** Phyllanthaceae **						
*Hieronyma alchorneoides* Allemão	x	x	x	-	-	-
** Polygonaceae **						
*Coccoloba alnifolia* Casar.	-	-	-	x	x	x
*Coccoloba warmingii* Meisn.	x	x	x	x	x	x
*Ruprechtia laxiflora* Meisn.	x	x	x	x	x	x
** Primulaceae **						
*Myrsine gardneriana* A.DC.	x	x	x	-	x	x
*Myrsine guianensis* (Aubl.) Kuntze	x	x	-	-	-	-
*Myrsine lineata* (Mez) Imkhan.	x	x	x	-	-	-
*Myrsine umbellata* Mart.	x	x	x	x	x	x
** Proteaceae **						
*Euplassa legalis* (Vell.) I.M.Johnst.	x	x	x	x	x	x
*Euplassa organensis* (Gardner) I.M.Johnst.	-	-	-	x	-	x
*Roupala montana* Aubl.	x	x	x	x	x	x
** Rosaceae **						
*Prunus myrtifolia* (L.) Urb.	x	x	x	x	x	x
** Rubiaceae **						
*Amaioua intermedia* Mart. ex Schult. & Schult.f.	x	x	x	x	x	x
*Cordiera concolor* (Cham.) Kuntze	x	x	x	x	x	x
*Faramea nigrescens* Mart.	x	x	x	x	x	x
*Ixora brevifolia* Benth.	x	x	x	x	x	x
*Psychotria vellosiana* Benth.	x	x	x	x	x	x
*Rudgea jasminoides* (Cham.) Müll.Arg.	-	-	-	x	x	x
** Rutaceae **						
*Metrodorea stipularis* Mart.	-	-	-	x	x	x
*Zanthoxylum fagara* (L.) Sarg.	-	-	-	x	x	x
*Zanthoxylum rhoifolium* Lam.	-	-	-	x	x	x
** Sabiaceae **						
*Meliosma sellowii* Urb.	-	-	-	x	x	-
** Salicaceae **						
*Casearia decandra* Jacq.	x	x	x	x	x	x
*Casearia obliqua* Spreng.	x	x	x	x	x	x
*Casearia sylvestris* Sw.	x	x	x	x	x	x
*Casearia ulmifolia* Vahl	x	x	x	-	-	-
*Xylosma ciliatifolia* (Clos) Eichler	-	-	-	x	x	x
*Xylosma prockia* (Turcz.) Turcz.	-	-	-	x	x	x
** Sapindaceae **						
*Allophylus semidentatus* (Miq.) Radlk.	-	-	-	x	x	x
*Cupania zanthoxyloides* Cambess.	x	x	x	x	x	x
*Matayba guianensis* Aubl.	x	x	x	x	x	x
*Matayba juglandifolia* Radlk.	-	-	-	x	x	x
** Solanaceae **						
*Solanum leucodendron* Sendtn.	-	-	-	x	x	x
*Solanum pseudoquina* A.St.-Hil.	-	-	-	x	x	x
** Styracaceae **						
*Styrax latifolius* Pohl	x	x	x	x	x	x
** Symplocaceae **						
*Symplocos celastrinea* Mart. ex Miq.	x	x	x	x	x	x
** Thymelaeaceae **						
*Daphnopsis brasiliensis* Mart. & Zucc.	-	-	-	x	x	x
*Daphnopsis fasciculata* (Meisn.) Nevling	x	x	x	x	x	-
*Daphnopsis utilis* Warm.	-	x	x	x	x	x
** Urticaceae **						
*Cecropia glaziovii* Snethl.	x	x	-	x	x	x
*Urera baccifera* (L.) Gaudich. ex Wedd.	-	-	-	x	x	x
** Vochysiaceae **						
*Qualea cordata* (Mart.) Spreng.	-	-	-	x	x	x
*Qualea dichotoma* (Mart.) Warm.	x	x	x	-	-	-
*Qualea multiflora* Mart.	-	-	-	x	x	x
*Vochysia magnifica* Warm.	-	-	-	x	x	x
*Vochysia tucanorum* Mart.	x	x	x	x	x	x

**Table 2.** Tree stand structure, diversity and species richness from two stands (B and C) in the Semideciduous Forest at the Parque Ecológico Quedas do Rio Bonito (PEQRB), municipality of Lavras, South of Minas Gerais State, Southeast Brazil. N ind = total number of individuals; N Species = species richness; N (tree ha^-1^) = number of individual per hectare; BA (m^2^ ha^-1^) = Basal area per hectare;Total BA = total basal area; *H*’ = Shannon-Weaver index (nats. indivídual^-1^); *J* = Pielou’s evenness index. A letter in the census of one stand followed by a different letter in the census of the another stand indicates significant difference in the comparisons. N ind was not significant different neither within nor among stands comparisons in all censuses. Total BA did not differ over censuses within each stand, but was different in all censuses comparisons among the two stands.

**Parameters**	**B**			**C**		
Censuses	2000	2005	2011	2001	2006	2011
N ind	1364^a^	1313 ^a^	1251 ^a^	1941 ^a^	1970 ^a^	1810 ^a^
N (tree ha^-1^)	1748.7	1683.3	1603.8	1702.6	1728.0	1587.7
BA (m^2^ ha^-1^)	19.02	20.03	21.23	24.52	25.81	26.94
Total BA	14.83 ^a^	15.62 ^a^	16.56 ^a^	27.95 ^b^	29.42 ^b^	30.71 ^b^
N Species	118 ^a^	115 ^a^	106 ^a^	157 ^b^	160 ^b^	157 ^b^
*H*’	3.97 ^a^	3.94 ^a^	3.86 ^a^	4.36 ^b^	4.36 ^b^	4.40 ^b^
*J*	0.83	0.83	0.82	0.86	0.85	0.87

**Table 3.** The dynamic tree components of two stands (B and C) in a Semi-deciduous Forest in the Parque Ecológico Quedas do Rio Bonito, municipality of Lavras, South of Minas Gerais State, Southeast Brazil. Dec. = decrease; Inc. = increase.

**Stands**:	**Stretch B**		**Stretch C**	
	**2000/2005**	**2005/2011**	**2001/2006**	**2006/2011**
Sampling:				
Number of plots	26	26	38	38
Number of trees:				
Initial	1364	1313	1941	1970
Final	1313	1251	1970	1810
Survival	1188	1128	1770	1631
Dead	176	187	171	339
Recruits	125	123	200	179
Mortality rate (%.year^-1^)	2.72	3.02	1.82	3.70
Recruitment rate (%.year^-1^)	1.98	1.63	2.11	1.71
Turnover rate (%.year^-1^)	2.35	2.45	1.97	2.71
Net change rate (%.year^-1^)	-0.75	-0.96	0.29	-1.67
Basal area:				
Initial	14.83	15.62	27.95	29.42
Final	15.62	16.56	29.42	30.71
Dead (m^2^)	1.83	1.85	2.15	3.38
Dec. survival (m^2^)	0.05	0.16	0.20	0.52
Recruits (m^2^)	0.32	0.42	0.51	0.90
Inc. survival (m^2^)	2.34	2.52	3.31	4.28
Loss rate (%.year^-1^)	2.41	2.45	1.63	2.52
Gain rate (%.year^-1^)	3.68	3.84	2.74	3.63
Turnover rate (%.year^-1^)	3.05	3.14	2.19	3.08
Net change rate (%.year^-1^)	1.04	1.17	1.02	0.86

**Table 4.** Dynamics of diameter classes of the tree component of stands B in a semi-deciduous forest in the Parque Ecológico Quedas do Rio Bonito, surveyed in the years 2000, 2005 and 2011. In the first line of the bale the captions mean: DBH (diameter at breast height); N: total number of individuals per class; D: number of dead trees per class; R: number of recruits per class and I (ingrowth) and O (outgrowth) are based on their absolute numbers; C. Poisson: Poisson counting comparing statistically ingrowth and outgrowth. In the second line of the table the captions mean: exp: expected frequency of the number of dead trees in each interval; % year^-1^: annual mean rate of mortality; N: Number of individuals per each category (D, O, R and I) and P: p-value of significance to Poisson Counting.

**DBH**	**N**			**D**			**O**	**R**	**I**	**C. Poisson**	
**(cm)**	**2000**	**2005**	**exp.**	**N**	**exp.**	% **year^-1^**	**N**	**N**	**N**	**Z**	**P**
≥05-10	867	789	834.583	133	128.027	3.27	73	125	3	4.29	<0.0001
>10-20	415	427	399.483	30	28.878	1.48	31	-	73	1.03	0.3008
>20-40	77	94	74.121	11	10.589	3.03	-	-	28	2.76	0.0057
>40-80	5	3	4.813	2	1.925	9.71	-	-	-	1.31	0.1890
	**2005**	**2011**									
≥05-10	789	686	748.739	134	127.162	3.65	90	116	4	5.66	<0.0001
>10-20	427	455	405.211	39	37.010	1.89	26	7	1	7.34	<0.0001
>20-40	94	101	89.203	14	13.286	3.17	2	-	22	0.96	0.3337
>40-80	3	4	2.847	0	0	-	-	-	1	0.79	0.4282

**Table 5.** Dynamics of diameter classes of the tree component of stand C in a semi-deciduous forest in the Parque Ecológico Quedas do Rio Bonito, surveyed in the years 2001, 2006 and 2011. In the first line of the bale the captions mean: DBH (diameter at breast height); N: total number of individuals per class; D: number of dead trees per class; R: number of recruits per class and I (ingrowth) and O (outgrowth) are based on their absolute numbers; C. Poisson: Poisson counting comparing statistically ingrowth and outgrowth. In the second line of the table the captions mean: exp: expected frequency of the number of dead trees in each interval; % year^-1^: annual mean rate of mortality; N: Number of individuals per each category (D, O, R and I) and P: p-value of significance to Poisson Counting.

**DBH**	**N**			**D**			**O**	**R**	**I**	**C. Poisson**	
**(cm)**	**2001**	**2006**	**exp.**	**N**	**exp.**	% **year^-1^**	**N**	**N**	**N**	**Z**	**P**
≥05-10	1103	1091	1113.229	111	112.029	2.09	92	200	2	11.41	<0.0001
>10-20	612	625	617.675	45	45.417	1.51	34	-	2	10.42	<0.0001
>20-40	213	229	214.975	13	13.121	1.25	5	-	34	2.22	0.0258
>40-80	13	14	13.121	2	2.019	3.28	-	-	3	0.41	0.6755
	**2006**	**2011**									
≥05-10	1091	936	993.54	233	212.186	4.53	93	167	12	6.6	<0.0001
>10-20	625	585	569.168	86	78.318	2.91	43	-	98	2.05	0.0395
>20-40	229	245	208.543	19	17.303	1.71	11	-	43	1.51	0.1256
>40-80	14	18	12.749	1	0.911	1.47	1	-	5	1.09	0.2717

**Table 6.** Dynamics of tree populations most abundant in the forest of the Parque Ecológico Quedas do Rio Bonito, municipality of Lavras, South of Minas Gerais State, Southeast Brazil. RG = regeneration guilds (following: Garcia 2012, Oliveira Filho et al. 2007). St = shade tolerants; Ld = light-demanding; Pion. = pioneer; N1, N2 and N3 = total number of trees in each one of the three inventories; and BA1, BA2 and BA3 = basal area in each one of the three inventories, respectively; D1 e D2 = rates of mortality in both analyzed periods; R1 and R2 = recruitment in both analyzed periods. ^1^ 10 most abundant ones in stand B and ^2^ in stand C, respectively.

**Species**	**Family**	**RG**	**N° trees**			**Rates**				**Basal area**		
			N1	N2	N3	D1	D2	R1	R2	BA1	BA2	BA3
*Amaioua intermedia* Mart. Ex Schult. & Schult.f. ^1^	Rubiaceae	Sb	112	121	136	0.90	0.50	2.42	2.79	0.65	0.83	1.04
*Copaifera langsdorffii* var. krukovii Dwyer ^1^	Fabaceae	Sb	89	89	90	0.68	0.68	0.68	0.67	1.28	1.39	1.47
*Croton echinocarpus* Müll. Arg. ^1^	Euphorbiaceae	Pion.	31	28	31	3.45	4.70	1.47	6.62	0.42	0.50	0.51
*Faramea latifolia* (Cham. & Schltdl.) DC. ^1^	Rubiaceae	St	36	36	34	1.13	1.72	1.13	0.59	0.37	0.40	0.39
*Myrsine umbellata* Mart. ^1^	Primulaceae	St	36	30	21	4.90	7.78	1.37	0.97	0.23	0.22	0.15
*Pera glabrata* (Schott) Poepp. Ex Baill. ^1^	Peraceae	Ld	61	64	65	0.66	0.63	1.61	0.94	1.25	1.51	1.69
*Protium widgrenii*Engl. ^1^	Burseraceae	Ld	68	67	67	0.89	1.53	0.60	1.53	0.67	0.74	0.81
*Psychotria vellosiana* Benth. ^1^	Rubiaceae	Ld	62	46	29	18.7	16.2	13.7	7.16	0.19	0.14	0.09
*Siphoneugena densiflora* O. Berg ^1^	Myrtaceae	St	80	75	75	1.81	2.23	0.53	2.23	0.39	0.42	0.47
*Tapirira obtusa* (Benth.) J.D.Mitch. ^1^	Anacardiaceae	Ld	67	64	52	2.18	5.20	1.28	1.18	0.80	0.87	0.76
*Cupania zanthoxyloides* Cambess. ^2^	Sapindaceae	Ld	40	42	40	2.63	2.50	3.58	1.54	0.24	0.24	0.27
*Eremanthus erythropappus* (DC.) MacLeish ^2^	Asteraceae	-	41	42	25	2.56	10.5	3.03	0	0.42	0.45	0.39
*Eugenia acutata* Miq. ^2^	Myrtaceae	St	54	56	57	0.75	0.35	1.47	0.71	0.52	0.59	0.65
*Miconia sellowiana* Naudin ^2^	Melastomataceae	Ld	110	114	67	2.08	12	2.78	2.18	0.90	0.78	0.58
*Myrcia splendens*: (Sw.) DC. ^2^	Myrtaceae	Ld	99	100	51	3.00	14.7	3.19	1.20	0.67	0.65	0.32
*Prunus myrtifolia* (L.) Urb. ^2^	Rosaceae	Ld	51	49	49	3.35	3.97	2.57	3.97	0.65	0.33	0.62
*Rudgea jasminoides* (Cham.) Müll. Arg. ^2^	Rubiaceae	St	42	49	52	3.58	2.57	6.50	3.72	0.21	0.25	0.28
*Siphoneugena densiflora* O. Berg ^2^	Myrtaceae	St	53	60	59	0.38	3.19	2.82	1.03	0.86	0.98	1.11
*Tapirira obtusa* (Benth.) J.D.Mitch. ^2^	Anacardiaceae	Ld	95	98	95	1.29	2.12	1.90	1.51	1.27	1.45	1.56
*Vochysia magnífica* Warm. ^2^	Vochysiaceae	Ld	98	89	79	1.90	2.35	0	0	1.61	1.86	2.34
